# Administration Timing Is the Best Clinical Outcome Predictor for Adalimumab Administration in Crohn's Disease

**DOI:** 10.3389/fmed.2019.00234

**Published:** 2019-11-01

**Authors:** Mauro Mastronardi, Margherita Curlo, Elisabetta Cavalcanti, Osvaldo Burattini, Renato Cuppone, Romina Tauro, Stefania De Santis, Grazia Serino, Pasqua Letizia Pesole, Elisa Stasi, Maria Lucia Caruso, Rossella Donghia, Vito Guerra, Pietro Giorgio, Marcello Chieppa

**Affiliations:** ^1^Department of Research, National Institute of Gastroenterology “S. de Bellis”, Bari, Italy; ^2^Department of Pharmacy, Faculty of Pharmacy and Medicine, University of Salerno, Fisciano, Italy

**Keywords:** Crohn's disease, biological agents anti-TNF, Adalimumab, clinical outcome, clinical remission

## Abstract

Biological intervention for Crohn's Disease (CDs) patients, mainly using anti-TNF antibodies, is often an efficient therapeutic solution. Nonetheless, data defining the administration timing to maximize the chances of clinical remission are lacking. The objective of this “real-life” retrospective study was to evaluate if early Adalimumab (ADA) administration (<12 months) was an efficient strategy to improve patients' clinical outcome. This single center study included 157 CD patients, of which 80 received the first ADA administration within the first 12 months from the diagnosis. After 1 year of therapy, clinical remission was observed in 50.32% of patients, mucosal healing in 37.58%. Clinical remission was observed in 66.25% of the early ADA administration patients vs. 33.77% of the late (>12 months) (*p* < 0.001); mucosal healing was observed in 53.75% of the early vs. 20.78% of the late (*p* < 0.001). Dose escalation was required for 30.00% of the early vs. 66.23% of the late (<0.01). In the early ADA administration group, 7.50% patients were considered non-responders at the end of the follow-up vs. 22.08% patients in the late administration group. These findings highlighted that early ADA administration (within 1 year of diagnosis) improves the clinical response and mucosal healing, and reduces the loss of response rate and need for dose escalation.

## Introduction

Crohn's disease (CD) is a severe, chronic and debilitating inflammatory disease involving the gastrointestinal wall of the entire digestive tract. The etiology of CD involves genetic and environmental factors, even if an immunological inflammatory component is always present. The presence of chronic active inflammation can induce the development of bowel damage, such as stenosis and fistula. Crohn's disease hallmarks include chronic diarrhea, abdominal pain, rectal bleeding, weight loss and, in affected children, growth may be impaired (1–3). The disease is characterized by recurring flare-ups alternating with periods of remission; both periods have a variable duration (2). In particular, over 50% of CD patients will develop penetrating lesions or stricture over long-term follow-up, dictating a need for surgical intervention (4). Previously, IBD patients endured a lack of effective treatment options, and patients with moderate-to-severe CD were often relegated to prolonged systemic corticosteroid therapy and surgery as their only options. Resection of the lesions is a crucial strategy to manage fibrostenotic or medically refractory disease, which has a negative impact on patients' postoperative morbidity and mortality rates, as well as quality of life (5).

Since the CD etiology is unknown, currently there are no preventive strategies. Several treatments are nowadays available for inducing and/or maintaining remission in CD, but most patients need lifelong medication. The introduction of biological agents targeting tumor necrosis factor α (TNF) has dramatically changed the medical approach to CD. The first FDA approved anti-TNF for Crohn's disease was Infliximab (IFX) introduced in 1998, the second was Adalimumab (ADA) in 2007 and, more recently, Golimumab and Certolizumab became available. According to the most recent European and American Guidelines (European Crohn's and Colitis Organisation—ECCO—and the American Gastroenterological Association—GA), conventional treatments include anti-inflammatory drugs (corticosteroids, aminosalicylates, immunosuppressants such as thiopurines and methotrexate), antibiotics, nutritional therapy, and surgery. Biologic agents, such as tumor necrosis factor alpha inhibitors (anti-TNF agents), are recommended in CD cases that are refractory, dependent or intolerant to conventional treatments, in relapsing patients, or in the early stages of the disease in patients showing factors predictive of disease severity (6, 7).

Several recent studies suggested that TNF blocking agents are effective in Crohn's disease. In particular, a meta-analysis of 19 clinical trials compared the effectiveness and safety of TNF blocking agents (Infliximab, Adalimumab, and Certolizumab) in the treatment of CD, showing that anti-TNF therapy is safe and significantly more effective than placebo (8). Furthermore, a meta-analysis involving 12,586 CD patients reported that thiopurine administration resulted in a 40% decrease in the first intestinal resection (9). Similarly, subgroup analysis of the ACCENT II and CHARM studies demonstrated that IFX and ADA maintenance therapy reduced the need for both hospitalization and surgery (10, 11).

It is currently debated whether an earlier start on biologic drugs may curtail long-term complications, such as strictures and fistulae. In *post-hoc* analysis of the CHARM and ADHERE trials, the authors reported a significant improvement of the remission rates in CD patients who started ADA within the first 2 years from the diagnosis compared with those starting after 5 years (12). However, different open label cohort studies failed to confirm the same effect of early anti-TNF therapy. Our study aims to fill the knowledge gap about the link between administration timing and clinical outcome.

## Materials and Methods

### Study Design

This single center case-series retrospectively evaluated Crohn's disease patients receiving Adalimumab between August 2008 and February 2016 at the Division of Gastroenterology and Digestive Endoscopy of the National Institute of Gastroenterology “S. de Bellis”, Castellana Grotte, Bari, Italy.

### Patients Population

Ethics Statement: The investigation has been conducted in accordance with the ethical standards, the Declaration of Helsinki and international guidelines, and has been approved by the authors' institutional review board. All patients provided written informed consent.

The following criteria were used for patients' selection: CD diagnosis by either endoscopy, histology or radiology (MRI) (within the established date of Aug 2008). CD was classified according to the Montreal Classification (13). All data were analyzed to identify factors predictive of the clinical outcome.

All consecutively enrolled adult patients (between 18 and 71 years old) with active Crohn's disease, treated with Adalimumab, were included. Adalimumab monotherapy was administered at the dose of 160/80 mg for the induction regimen and 40 mg every other week for maintenance. Dose escalation was defined as increasing the frequency to weekly injections.

The primary endpoint was: Mucosal Healing (MH), defined according to the Simple Endoscopic Score for Crohn's Disease (SES-CD), a simple, reproducible, and easy-to-use endoscopic scoring system for Crohn's disease, based on ulcer size, ulcerated and affected surfaces and stenosis (a SES-CD score <2 means mucosal healing). The SES-CD score was assessed on each endoscopic evaluation from the first one to the end of the follow-up; -percentage of patients in deep remission calculated as concomitant clinical remission (HB score < 5), mucosal healing (SES-CD < 2) and C-reactive protein (CRP) in the reference range-safety (reported adverse events, laboratory tests) (14, 15). The secondary endpoints were: -clinical remission 52 weeks from the beginning of ADA administration defined according to the Harvey Bradshaw Index -HBI, a simple index of Crohn's disease activity based on the evaluation of general well-being, abdominal pain, number of liquid, or soft stools per day, abdominal mass and complications (an index score < 5 meaning remission); -steroid-free clinical remission 52 weeks from the start of the treatment and during the follow-up.

We also evaluated the clinical response (3 points or more from the baseline score HB) and the endoscopic improvements, defined as a reduction of the SES-CD score by more than 50% compared to baseline. Outcome analysis consisted of evaluating clinical and bio-humoral parameters every 3 months. The evaluation of clinical remission and mucosal healing, as well as of the secondary endpoints, was performed 12 months from the start of the therapy.

### Statistical Analysis

Continuous data were expressed as mean and standard deviation if normally distributed, as median and interquartile range (IQR) otherwise. Comparisons between values at the beginning and at the end of the study were performed with paired *t*-test for normally distributed variables, or Wilcoxon test for paired data.

Another aim was to evaluate predictors of a SES-CD. This score was classified as <2 and ≥2 and its value at the end of the study was the dependent variable of a logistic regression model. Predictors tested in the univariate model were: age class, gender, smoking habit, months from the diagnosis, dose escalation, steroid therapy, steroid dependency, steroid resistance, other therapies, type and site of disease, number of cycles and duration of steroid therapy, calprotectin at the beginning of the study, ferritin at the beginning, CRP at the beginning, albumin at the beginning.

A multivariate model was also built to evaluate predictors independently related to SES-CD; all variables were included in the model and then selected using the stepwise procedure. The final model included age class, sex and smoking habit as adjustment variables, together with those variables selected by the stepwise procedure.

In both models, to evaluate the effect of adjusting variables Type 3 analysis *p*-values were reported; to evaluate the statistical significance of the model the chi-square score was used, while the fitting of the models was assessed by considering the Hosmer and Lemeshow statistic (HL chi-square, a *p* > 0.05 suggests an adequate fitting).

A *p* < 0.05 was selected as statistically significant. All the analyses were performed with SAS 9.4 for PC.

## Results

### Cohort Characteristics

One hundred fifty-seven patients (mean age 34.99 years, 68.15% males, 36.31% smokers) were enrolled in the study and followed up for a median time of 50 (6–102) months. Demographic and clinical characteristics of the patients enrolled are summarized in Table 1. The endoscopic evaluation was performed in all patients at a mean time of 12.5 months (range 10.8–16.4 months) from the beginning of the therapy. A second endoscopy was performed at a mean of 13.4 months from the first endoscopic evaluation (range 11.2–16.9 months). Disease distribution was 48 in the ileum (30.77%), 81 ileocolic (51.92%), and 27 colic (17.31%).

**Table 1:** Clinicpathologic features of enrolled patients.

	***n* = 157**
Follow-up (months)	
Median (range)	50 (6–102)
Age (years)	
Mean (SD)	34.99 (14.36)
Median (range)	33.00 (12.00–74.00)
Gender (Male) (%)	107 (68.15)
Smokers (%)	57 (36.31)
Time from diagnosis to start ADA (months)	
Mean (SD)	32.48 (43.30)
Median (range)	12 (1–265)
Type of disease (%)–Montreal classification	
Inflammatory	61 (38.85)
Stricturing	47 (29.94)
Penetrating	49 (31.21)
Location of disease *N* (%)–Montreal classification	
L1 Ileal	48 (30.77)
L2 Colic	27 (17.31)
L3 Ileocolic	81 (51.92)
Steroid therapy (%)	143 (91.08)
Steroid therapy, cycles	
Mean (SD)	2.64 (2.62)
Median (range)	2 (0–16)
Steroid therapy, total duration (weeks)	
Mean (SD)	30.08 (29.48)
Median (Range)	20 (0–170)
Steroid resistant patients (%)	4 (2.56)
Steroid dependent patients (%)	110 (70.51)

The disease phenotypes at diagnosis were inflammatory in 61 (38.85%) patients, stricturing in 47 (29.94%) and penetrating in 49 (31.21%) patients.

The majority of the observed patients 143 (91.01%) received at least one systemic steroids cycle before starting Adalimumab (with a median (IQR) duration equal to 20 weeks). However, not all patients responded to corticosteroid therapy. In our study, four (2.56%) of patients failed to respond to the initial treatment with steroids, while 110 (70.51%) of patients may be considered to be steroid-dependent (Table 1). Moreover, 75 patients received treatments other than steroids, including azathioprine 43 (27.3%), Infliximab 21 (13.3%), a combination of azathioprine and Infliximab 6 (3.8%), and antibiotics 5 (3.2%) (data not shown).

### Adalimumab on Clinical Outcomes

The entire cohort of 157 patients described was treated using Adalinumab, the clinical outcomes was evaluated and analyzed. Of note, the administration of Adalimumab was withdrawn for a lack of response in four patients, and in one patient due to adverse events (severe psoriasis). Clinical remission was achieved in 79 (50.32%) patients at 12 months following the beginning of ADA administration, clinical response was observed in 55 (35.03%) patients (Table 2). Steroid-free remission was observed in 98 (62.42%) of the patients in clinical remission or clinical response. Mucosal healing was achieved in 59/157 patients (37.58%) treated with Adalimumab.

**Table 2:** Efficacy of ADA on clinical outcomes in patients with a disease duration of <12 months vs. more than 12 months.

		**Administration ADA**		
	**At 52 week**	**<12 months**	**≥12 months**	*p-*value^*^
	***n* = 157**	***n* = 80**	***n*: 77 (%)**	
Clinical remission (%)	79 (50.32)	53 (66.25)	26 (33.77)	<0.001
Clinical response (%)	55 (35.03)	21 (26.25)	34 (44.16)	0.02
Deep remission (%)	54 (34.39)	39 (48.75)	15 (19.48)	<0.001
Endoscopic improvement (%)	42 (26.75)	29 (36.25)	13 (16.88)	0.006
Mucosal healing (%)	59 (37.58)	43 (53.75)	16 (20.78)	<0.001
Dose escalation (%)	75 (47.77)	24 (30.00)	51 (66.23)	<0.001
Steroid-free remission (%)	98 (62.42)	71 (88.75)	27 (35.06)	<0.001
Non-responder (%)	23 (14.65)	6 (7.50)	17 (22.08)	0.01^$^

* *Chi-square test*;

$ *Fisher's exact test*.

At 52 weeks 54 (34.39%) patients obtained a deep remission and, at the same time, endoscopic improvement was detected in 42 (26.75%) patients. Among patients with endoscopic improvement, 32/42 (76.19%) patients achieved clinical remission and 10/42 (23.80%) clinical response. Finally, only 23 (14.65%) patients were complete non-responders (Table 2).

At the end of the follow-up, among patients that obtained clinical response, 11/55 had clinical remission and 10/42 patients with endoscopic improvement had shifted to mucosal healing; in total 90/157 (57.32%) patients achieved in clinical remission and 69/157 (43.94%) patients were in mucosal healing. At the same time, 23/157 (14.65%) were complete non-responders, among them, 14/157 (8.92%) underwent intestinal resection (data not shown).

Dose escalation (defined as an increase in the selected ADA dose to 40 mg every week instead of every 2 weeks) was required in 75/157 (47.77%) cases (Table 2).

Clinical assessment, performed at the end of the follow-up for the whole cohort of 157 patients, showed significantly improved values compared to baseline, especially for those related to inflammation (CRP, HBI, SES-CD) (Table 3). Both the logistic regression model on single factor and the multiple logistic regression model on all factors identified the following factors as being significantly associated with unsuccessful clinical remission: age, number of cycles of steroid therapy, duration of steroid therapy, dose escalation, months from diagnosis, ileocolic disease, and previous anti-TNF therapy. Furthermore, factors significantly associated with unsuccessful mucosal healing were the number of cycles of steroid therapy, duration of steroid therapy, and dose escalation (Table 4).

**Table 3:** Change of clinical outcome parameters from baseline to the last visit.

**Parameters**	**Baseline**	**Last observation**	*p*-value^*^
CRP level <5 g/L			<0.0001
Mean (SD)	39.80 (37.27)	13.44 (23.93)	
Median (range)	30 (2–175)	4 (1–170)	
Ferritin level <30 mg/dL			<0.0001
Mean (SD)	17.22 (10.49)	31.39 (14.78)	
Median (range)	15 (2.1–55)	31 (2.8–88)	
HBI score			<0.0001
Mean (SD)	13.59 (4.06)	7.78 (5.18)	
Median (range)	14 (6–28)	6 (1–26)	
SES-CD			<0.0001
Mean (SD)	13.67 (5.79)	7.00 (5.62)	
Median (range)	13 (0–42)	5 (0–23)	
Fecal Calprotectin μg/g			<0.0001
Mean (SD)	404.35 (220.55)	228.56 (328.66)	
Median (range)	376.50 (16–1,239)	112 (22–3,313)	
Weight (Kg)			<0.0001
Mean (SD)	65.40 (13.00)	69.15 (13.73)	
Median (range)	65 (41–113)	67 (46.136)	

* *Wilcoxon signed-rank test*.

**Table 4:** Logistic regression model of clinical remission, and of mucosal healing on single factor.

**Variable**	**Odds Ratio**	**se(OR)**	**95% Cl**	***p*-value**
**Clinical remission**				
Age	0.97	0.01	0.95–0.99	0.02
Number of cycles of steroids	0.67	0.07	0.55–0.82	<0.001
Duration of steroid treatment	0.96	0.01	0.95–0.98	<0.001
Dose escalation	0.32	0.11	0.17–0.62	0.001
Months from diagnosis	0.98	0.005	0.97–0.99	0.001
Ileocolic disease	1.15	0.20	0.81–1.64	0.44
Previous anti TNF	0.21	0.12	0.06–0.65	0.007
**Mucosal healing**				
Number of cycles of steroids	0.67	0.08	0.54–0.85	0.001
Duration of steroid treatment	0.97	0.01	0.95–0.99	0.001
Dose escalation	0.40	0.14	0.20–0.78	0.008
**Multiple Logistic Regression Model of Clinical Remission and of Mucosal Healing on All Factors**				
**Clinical remission**				
Age	0.98	0.01	0.95–1.00	0.10
Number of cycles of steroids	0.93	0.21	0.59–1.44	0.74
Duration of steroid treatment	0.98	0.02	0.94–1.02	0.27
Dose escalation	0.50	0.19	0.23–1.05	0.07
Months from diagnosis	1.00	0.01	0.98–1.01	0.82
Ileocolic disease	1.20	0.25	0.80–1.82	0.37
Previous anti TNF	0.44	0.29	0.12–1.57	0.21
**Mucosal healing**				
Number of cycles of steroids	0.78	0.17	0.51–1.21	0.28
Duration of steroid treatment	0.99	0.02	0.95–1.03	0.55
Dose escalation	0.55	0.20	0.27–1.14	0.11

*OR, Odds Ratio; se(OR), standard error of OR*.

### Early Disease Population

Short duration of the disease seems to be correlated to a better outcome, therefore we performed a sub-analysis comparing patients treated with ADA <12 months following the disease diagnosis (80/157) vs. more than 12 months (77/157). The main baseline characteristics of the patients enrolled are summarized in Table 5. Patients with a shorter disease duration were younger (31.60 years vs. 38.51 years, *p* ≤ 0,002), and had taken lower doses of steroids or previous anti TNF (1.51 mean steroid cycle vs. 3.82 *p* ≤ 0.0001 and 3.75% vs. 22.08% previous use of anti TNF *p* ≤ 0.001), compared to patients with a disease duration >12 months. No differences were found regarding smoking habit, baseline disease activity, disease distribution, and behavior. Moreover, 80/157 (50.95%) patients started ADA treatment within 12 months (average time 6.17 months) and 77/157 (49.04%) patients after 12 months (average time 59.82 months) from diagnosis of CD. Besides, differences for CRP, ferritin, fecal calprotectin, albumin, SED-CD, and HBI levels between patients with disease duration <12 months and patients with disease duration >12 months are reported in Table 5.

**Table 5:** Baseline characteristics patients treated with ADA with a disease duration of <12 months vs. more than 12 months.

	**Administration ADA**		
	**<12 months**	**>12 months**	*p*-value^*^
	**(*n* = 80)**	**(*n* = 77)**	
Age (years)			0.002
Mean (SD)	31.60 (13.42)	38.51 (14.55)	
Median (range)	28 (12–64)	37 (15–74)	
Sex (Male) (%)	54 (67.50)	53 (68.83)	0.86^∧^
Smokers (%)	26 (32.50)	31 (40.26)	0.31
Montreal classification–Behavior (%)			0.44
Inflammatory	35 (43.75)	26 (33.77)	
Stricturing	22 (27.50)	25 (32.47)	
Penetrating	23 (28.75)	26 (33.77)	
Montreal classification–Disease location (%)			0.24
L1	20 (25.32)	28 (36.36)	
L2	13 (16.46)	14 (18.18)	
L3	46 (58.23)	35 (45.45)	
ADA started, months			<0.0001
Mean (SD)	6.17 (3.24)	59.82 (48.49)	
Median (range)	6 (1–12)	46 (13–265)	
Steroid cycle			<0.0001
Mean (SD)	1.51 (0.95)	3.82 (3.23)	
Median (range)	1 (0–6)	3 (0.16)	
Previous anti TNF (%)	3 (3.75)	17 (22.08)	0.001^∧^
CRP, mean (SD)			0.87
Mean (SD)	39.51 (36.48)	40.09 (38.31)	
Median (range)	32 (3–175)	25 (2–165)	
Ferritin, mean (SD)			0.21
Mean (SD)	16.15 (10.03)	18.32 (10.90)	
Median (range)	14.50 (2.30–45.00)	17.00 (2.10–55.55)	
Fecal Calprotectin, mean (SD)			0.03
Mean (SD)	437.99 (220.85)	368.89 (216.06)	
Median (range)	389 (16–1231)	335 (45–1239)	
Albumin, mean (SD)			0.17
Mean (SD)	2.90 (0.43)	3.01 (0.46)	
Median (range)	3 (1.7–3.8)	3 (1.9–4.1)	
SES-CD, mean (SD)			0.003
Mean (SD)	15.11 (6.22)	12.17 (4.90)	
Median (range)	15 (7–42)	11 (0–25)	
HBI, mean (SD)			0.53
Mean (SD)	13.77 (3.97)	13.40 (4.17)	
Median (range)	14 (7–28)	13 (6–28)	

* *Wilcoxon rank-sum (Mann-Whitney) test*;

∧ *Chi-square test*.

**Figure 1:**
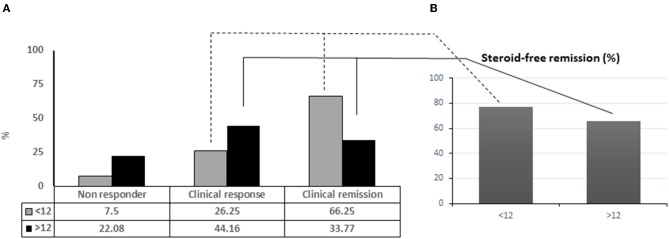
Adalinumab treated patients achieving complete remission in relation with disease duration **(A)**. Percentage of patients under steroid free remission **(B)**.

Among all patients in deep remission (54/157), 39 (48.75%) were included in the group with disease duration <12 months vs. 15 (19.48%) with disease duration >12 months. The clinical remission rate was significantly superior for patients with disease duration <12 months (66.25%) vs. patients with disease duration >12 months (33.77%) (*p* ≤ 0,001), and also better for the overall population (50.32%) (Figure 1A) (Table 2). Significant clinical response was observed in patients with disease duration >12 months (*p* = 0.02) to prove that adalimumab represents an effective and well-tolerated therapeutic option. Mucosal healing was significantly more frequent in patients with a disease duration <12 months, compared to patients with a disease duration >12 months (*p* < 0.001). At the end of the follow-up, almost all patients with a disease duration <12 months were characterized by endoscopic improvement 72/80 (43/80 MH and 29/80 endoscopic improvement) compared to patients with disease duration >12 months 29/77 (16/77 with MH and 13/77 with endoscopic improvement). Patients with a disease duration of <12 months achieved significant corticosteroid-free remission (*p* < 0.001) (Figure 1B). Dose escalation of ADA was obtained as increased frequency of weekly injections was successful in 75/157 (47.77%), 24/80 (30.00%) patients with a disease duration <12 months and 51/77 (66.23%) patients with a disease duration >12 months (Table 2). 2/80 patients (2.5%) treated with early ADA administration needed surgical resection at the end of the follow-up, compared to 12/77 (15.5%) of patients with late ADA treatment (data not shown).

### Anti TNF Naïve vs. Non-naïve

Of the 157 patients within the study, 137 (87.26 %) were naïve and 20 (12.73%) patients experienced anti-TNF treatment. Patients with disease duration <12 months were anti-TNF naïve 77/80 (96.25%) and 3/80 experienced (3.75%), patients with disease duration >12 months were anti-TNF naïve 60/77 (77.92%) and 17/77 experienced (22.07%). In particular, 68/157 (51 < 12 + 24 > 12) naïve patients achieved the clinical remission at 12 months compared with 4 (2 < 12 + 2 > 12) experienced patients. Therefore, we compared clinical remission, mucosal healing and dose escalation to determine the clinical outcome in both naive and (non-naïve) experience patients (Table 6). The rates of clinical remission were significantly higher in naïve patients with disease duration <12 months (66.23% vs. 40.00%, respectively; *p* = 0.002), while no significant difference was observed among experienced patients with duration disease <12 and >12 months (*p* = 0.09). A similar trend was observed for mucosal healing and dose escalation between naïve and experienced patients (Table 6).

**Table 6:** Clinical outcomes at 12 mo–patients previously treated with anti-TNF.

	**Administration ADA**					
	**Naive**			**Experienced**		
	**<12 months**	**>12 months**	***p*-value**	**<12 months**	**>12months**	***p*-value**
	**(*n* = 77)**	**(*n* = 60)**		**(*n* = 3)**	**(*n* = 17)**	
Clinical remission	51 (66.23)	24 (40.00)	0.002^∧^	2 (66.67)	2 (11.76)	0.09^§^
Mucosal healing	42 (54.55)	15 (25.00)	<0.001^∧^	1 (33.33)	1 (5.88)	0.28^§^
Dose escalation	23 (29.87)	38 (63.33)	<0.001^∧^	1 (33.33)	13 (76.47)	0.20^§^

∧ *Chi-square test*.

§ *Fisher's exact test*.

## Discussion

Since the introduction of biological agents, treatment strategies, able to induce and maintain remission and mucosal healing for CD patients, have dramatically improved (16). While there can be no doubts about their efficiency, an open discussion is still required to identify the most effective administration timing to achieve long term remission rates. The aim of our single-center retrospective analysis was to evaluate the real life efficacy of Adalimumab in patients with CD, to identify factors predictive of clinical outcome and to fill the knowledge gap regarding administration timing and clinical outcome. Although, there are several other similar real-life cohorts published, our study represents a real-life study of adalimumab in a single-center retrospective cohort of Italian patients with Crohn's disease.

In our study, 12 months after the beginning of ADA therapy, clinical remission was observed in more than half of the treated patients (79/157; 50,32%) with further improvement in the performance (90/157; 57,3%) at the end of the follow-up. This response rate is substantially equivalent to those reported by other authors (1, 3, 16–20). Furthermore, the percentage of patients with mucosal healing (37.58%) at 52 weeks raised to 43.9% at the end of the follow-up. The endoscopic data was obtained by performing at least two endoscopic evaluations during the follow-up, the first of them at 12.5 months from the start of the treatment. Our data offer a solid assessment of the treatment efficiency 12 months after the beginning of the Adalimumab administration.

It is crucial to underline that our data support the importance of an endoscopic evaluation at 52 weeks for a correct evaluation of the treatment efficiency.

Results previously published by Song et al. demonstrated the efficacy and safety of Adalimumab for Crohn's disease (21, 22). Furthermore, our data confirm what was previously reported about clinical remission and mucosal healing rates at 6, 12, and 24 months from the beginning of the treatment (18). Our multivariate analysis identified dose escalation, intervention timing later than 12 months from the diagnosis and previous treatment with an anti-TNF as prognostic factors negatively related to clinical remission achievement.

Unsurprisingly, an inflammatory phenotype and a short disease duration was associated with a higher mucosal healing rate (18). Adalimumab efficiency was negatively but significantly affected by longer disease duration and presence of strictures (23). These data, as recently published by Miyoshi et al. (24) suggested that treatment with this agent in the early stages of the disease may improve the clinical outcome, likely by preventing fibrosis development and, consequently, the need for surgeries (25–27). Although our understanding of fibrogenesis in CD continues to evolve, we believe that early administration of anti-TNF may block or attenuate the cascade of events leading to the fibrogenic process. Several TNF mediated mechanisms could occur, including epithelial tight junction disassembly, causing increased intestinal permeability and, consequently, an increased subepithelial exposure to bacterial antigens (25). Furthermore, fibroblasts, vascular endothelial growth factors, and endothelial permeability may have a pivotal role in the amplification of the inflammatory cascade (28). In light of our data, it is tempting to speculate that early control of gut inflammation is critical to prevent fibrostenotic intestinal injury previously described as a major factor leading to poor patient outcomes (29). Furthermore, the higher steroid-free remission (56.8%) and mucosal healing rates (43.9%) reported in the SONIC trial (30), enrolling biologic and immunosuppressant naïve CD patients with a short disease duration (median 2.3 years), contribute to indirectly support our evidence.

In routine clinical practice, a second anti-TNF drug is used when a first one has failed, regardless of whether patients are primary non-responders, secondary non-responders, or intolerant. Unsurprisingly, the meta-analysis results published in a systematic review by Gisbert et al. demonstrated that the efficacy of switching the anti-TNF agent in CD patients largely depends on the reason for switching (31). As the onset of fibrotic areas has an inverse correlation with the success rate of anti-TNF treatment, it seems clear that the administration timing should be among the most important factors for ADA-mediated clinical remission. Our multivariate analysis showed that dose escalation was a negative prognostic factor for both clinical remission and mucosal healing. Some clinical trials demonstrated that in Crohn's disease, Adalimumab dose escalation to 40 mg weekly was effective for managing secondary loss of response, allowing more patients to maintain clinical remission (32, 33). A recently published prospective reported that Adalimumab 80 mg administered weekly seems to be well-tolerated and may be effective in inducing clinical remission in CD patients previously treated with lower Adalimumab doses (34, 35). Our data for dose escalation for secondary loss of response during maintenance therapy (in patients with successful primary response) was 47.77%, in line with previous findings.

Although limited by potential bias due to the patients' prior treatment history, once enrolled in our clinical protocol, all patients were scored on identical biomarkers, radioscopic and endoscopic parameters. Furthermore, even if results were obtained through a retrospective study of early vs. late ADA administration, no differences were detected between the two groups of patients in terms of inflammation, calprotectin, endoscopic, and clinical index. Of note, the outcome was similar for non-smokers vs. smokers, suggesting that this factor was not relevant in the present study.

Patients in the early ADA-administration had a lower risk of dose escalation compared with late patients 12 months following biologic drug initiation (30.00% vs. 66.23%) and a lower risk of discontinuing or switching treatment, compared with 12 months after biologic initiation (6/80 vs. 17/77). Thus, a top-down approach to anti-TNF therapy may avert secondary loss of response in some patients.

Finally, remission rates were greater in naïve to anti TNF compared to non-naïve (experienced), but this may be mostly a consequence of an earlier intervention timing in naïve patients.

In conclusion, even considering the limitation of the present study consisting in a single-center retrospective cohort of Italian patients with Crohn's disease our data indicate that Adalimumab is a valid therapeutic option for the management of patients with moderate to severe Crohn's disease. Administration timing is a crucial factor, predictive of clinical outcome, indicating that ADA treatment should begin within the first year following the CD diagnosis. Although no acknowledged consensus has been reached in regard to the optimal timing for the administration of biological drugs in IBD, this study supports the view that introducing ADA treatment during the “window period,” when structural alterations of the bowel are still not evident, may have a positive impact on the clinical history of the disease.

## Data Availability Statement

The datasets generated for this study are available on request to the corresponding author.

## Ethics Statement

The studies involving human participants were reviewed and approved by Comitato Etico Istituto Tumori Giovanni Paolo II Bari–Prot. n. 42 del CE De Bellis 27/06/2017. The patients/participants provided their written informed consent to participate in this study.

## Author Contributions

MM, MCu, OB, RC, ES, RT, and PG: assembly of tissue and data, study design, and wrote the manuscript. MM and MCh: conceived of the study and editing of the manuscript. MLC: histological evaluation. MM, SD, EC, GS, PP, RD, and VG: data interpretation, evaluation. All authors critically reviewed the manuscript and gave final approval for publication.

### Conflict of Interest

The authors declare that the research was conducted in the absence of any commercial or financial relationships that could be construed as a potential conflict of interest.
